# Microstructural Evolution and Competing Deformation Mechanisms in Aerospace Titanium Alloys: A Review

**DOI:** 10.3390/ma19132816

**Published:** 2026-07-02

**Authors:** Xin Xie, Yisong Peng, Weihe Xu, Xue Cui, Tongqi Zhang, Zhisheng Nong

**Affiliations:** School of Materials Science and Engineering, Shenyang Aerospace University, Shenyang 110136, China; x15770895124@163.com (X.X.); 18945719770@163.com (Y.P.); 13889122783@163.com (W.X.); a15902460336@163.com (T.Z.); nzsfir@163.com (Z.N.)

**Keywords:** titanium alloys, microstructure control, slip-twinning-transformation interplay, ω phase, mechanism competition

## Abstract

**Highlights:**

- The effects of lamellar, equiaxed, and bimodal microstructures on strength, ductility, and fatigue are summarized.
- The deformation mechanisms including slip, twinning, stress-induced phase transformation, and ω-related processes are reviewed.
- The competition and synergy among deformation mechanisms under microstructural constraints are emphasized.

**Abstract:**

Aerospace load-bearing components require materials that exhibit high specific strength, excellent fatigue resistance, and superior environmental adaptability. Titanium alloys are indispensable for aerospace applications because of their exceptional mechanical properties, particularly their outstanding high specific strength, and their peak mechanical strength is typically achieved through solution heat treatment followed by artificial aging. This review systematically summarizes recent advances in the compositional design, microstructural evolution, and critical microstructure–property relationships of aerospace titanium alloys. It further highlights intrinsic effects of alloying elements on phase stability, dislocation behavior, and phase transformation pathways, and analyzes how lamellar, equiaxed, and bimodal microstructures regulate dislocation transfer, local strain partitioning, and damage evolution. The interactions and competition among deformation and phase-transformation mechanisms, including slip anisotropy, deformation twinning, stress-induced phase transformations, and ω-related processes, are critically assessed. However, unresolved challenges remain in quantitatively characterizing multi-mechanism coupling and local heterogeneity. To address these challenges, this review elucidates the transition rules of dominant mechanisms across different microstructures and proposes a high-precision digital composition–microstructure–property mapping framework to facilitate predictive and service-oriented alloy design.

## 1. Introduction

The selection of structural materials for aerospace applications is strongly constrained by complex service conditions. Compared with conventional engineering structures, load-bearing components in aero-engines and airframes are subjected to the combined effects of cyclic loading, temperature fluctuations, and aggressive environmental conditions. Consequently, aerospace materials must achieve an optimal balance among specific strength, fatigue resistance, thermal stability, creep resistance, and environmental adaptability [1,2,3,4,5]. In response to these demanding requirements, several representative material systems have been developed for aerospace applications. These material systems include aluminum alloys, high-strength steels, titanium alloys, nickel-based superalloys, and composite materials. Each material system exhibits distinct advantages and inherent limitations. Aluminum alloys possess low density but exhibit limited high-temperature capability. High-strength steels provide excellent strength but limited lightweighting potential. Nickel-based superalloys are well suited for high-temperature applications but exhibit poor lightweighting capability. Composite materials remain limited by challenges associated with joint reliability and performance under complex service conditions [6,7].

**Figure 1:**
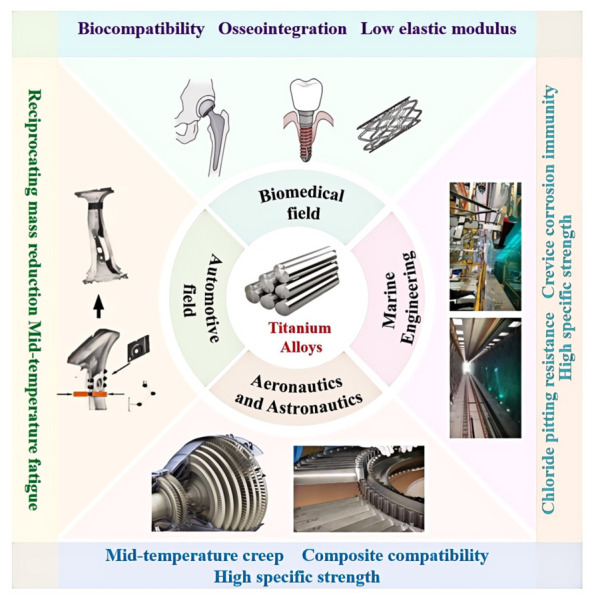
Schematic of titanium alloy application sectors [16,17,18,19].

Figure 1 schematically summarizes the application fields of titanium alloys. Owing to their low density, high specific strength, excellent corrosion resistance, and a favorable combination of mechanical properties within the moderate-temperature regime, titanium alloys offer outstanding advantages across multiple sectors [8,9,10]. In the aerospace sector, titanium alloys are widely used in load-bearing airframe structures. They are also extensively applied in cold-section and intermediate-temperature aero-engine components, including fan blades, compressor blades, casings, and critical connecting structures. These components require sufficient mechanical strength while satisfying stringent lightweighting requirements [11,12]. In addition, they must maintain excellent fatigue resistance, microstructural stability, and damage tolerance under long-term cyclic loading and moderate-temperature service conditions [13,14,15]. Therefore, the advantages of titanium alloys in aerospace applications do not arise from the exceptional performance of a single property alone. Instead, their value lies in achieving an optimized balance among lightweighting capability, load-bearing performance, and long-term service reliability.

The mechanical performance of titanium alloys is critically influenced by manufacturing routes and strengthening mechanisms [20]. Conventional thermomechanical processing together with additive manufacturing techniques enables tailored microstructures through controlled grain size, phase morphology, and crystallographic texture. The resulting mechanical properties are governed by multiple strengthening mechanisms, including solid-solution strengthening, precipitation hardening, and grain refinement. Furthermore, the long-term reliability of titanium alloys is closely associated with the stability of the surface TiO_2_ passive film, which can be degraded by elevated temperatures, mechanical damage, or aggressive species, thereby compromising corrosion resistance [21]. In particular, during hot forging, the TiO_2_ layer is susceptible to degradation, and an optimal temperature range must be maintained to balance oxidation resistance and formability. Temperature variations in hot processing not only affect oxide layer growth and spallation but also influence subsequent machining behavior and surface finish, as excessive oxidation can induce surface hardening and tool wear.

The microstructural characteristics of titanium alloys, which are intimately linked to both composition and processing as discussed above, ultimately originate from the differential stability of the α and β phases and their associated metastable derivatives. Pure titanium exhibits a hexagonal close-packed (HCP) α phase at ambient temperature and transforms into a body-centered cubic (BCC) β phase at elevated temperatures. Alloying elements primarily regulate the α/β phase fraction and phase stability by modifying the β-transus temperature, thereby enabling a wide range of attainable microstructures [22]. From an engineering perspective, titanium alloys are generally classified into α-type, near-α, α + β, and β-type/metastable β-type alloys. This classification is determined by the dominant phase fraction at room temperature and the intrinsic stability of the β phase [23]. α-type and near-α alloys consist predominantly of the α phase, which provides excellent high-temperature strength and creep resistance. α + β alloys achieve a favorable balance between strength and ductility through tailoring the size, morphology, and volume fraction of the α and β phases. In contrast, β-type and metastable β-type alloys exhibit high β-phase stability, and their subsequent microstructural evolution is strongly influenced by β retention and subsequent phase transformations. During the β→α transformation, particularly under non-equilibrium conditions, metastable phases such as α′, α″, and ω can readily form. These metastable phases can significantly influence mechanical properties and actively participate in α-phase nucleation and the evolution of the surrounding microstructure [24,25,26].

The design of titanium alloys is fundamentally constrained by the need to balance strength, ductility, thermal stability, and service reliability. Strength enhancement in titanium alloys, as outlined above, inevitably increases resistance to dislocation motion and consequently reduces ductility. Improving high-temperature stability can extend the allowable service temperature range. However, such improvements may compromise processability and lightweight capability. Transformation-induced plasticity (TRIP) or twinning-induced plasticity (TWIP) mechanisms can be introduced through metastable β-phase design. These mechanisms provide an effective route for the simultaneous enhancement of strength and ductility. Nevertheless, they may also introduce challenges associated with microstructural stability and long-term service reliability [27,28,29,30,31]. The mechanical response of titanium alloys is governed by the interactions among alloy composition, microstructure, processing history, and deformation behavior. Slip, twinning, and phase-transformation mechanisms interact strongly across different compositional systems and microstructural configurations. These mechanisms collectively govern local strain partitioning, work-hardening behavior, and susceptibility to crack initiation [32,33,34,35,36]. Microstructural characteristics, including grain size, lath thickness, interface structure, crystallographic texture, and local heterogeneity, further influence the activation sequence and relative contributions of these deformation mechanisms [37,38].

This review systematically summarizes the composition–microstructure–property relationships in aerospace titanium alloys, with particular emphasis on deformation mechanisms and their dependence on microstructural characteristics. The specific objectives of this review are as follows: First, the effects of alloying elements and typical microstructures (lamellar, equiaxed, and bimodal) on mechanical response and damage evolution are examined, and the associated property trade-offs are discussed. Second, the interactions among multiple deformation mechanisms-including slip anisotropy, solute-controlled dislocation motion, deformation twinning, stress-induced phase transformation, and the role of the ω phase-are critically assessed. Finally, current challenges and future research directions are discussed, together with the need for predictive approaches to titanium alloy design.

## 2. Methods

The literature reviewed in this work was identified through systematic searches of the Web of Science and Scopus databases using keywords related to titanium alloys, microstructure, deformation mechanisms, and mechanical properties. Additional relevant studies were identified by cross-referencing the bibliographies of retrieved articles. Only peer-reviewed journal articles published in English were considered, while conference proceedings and book chapters were excluded. No specific percentile or quartile-based inclusion criterion was adopted for database searches. After initial screening, the final reference list was selected based on the significance of contributions to the understanding of microstructural evolution and deformation mechanisms in aerospace titanium alloys, with priority given to high-quality publications from mainstream journals in the fields of materials science and metallurgy.

## 3. Alloying and Microstructure

### 3.1. Alloying Element Effects

Table 1 summarizes the mechanical properties of representative titanium alloys. The mechanical properties of titanium alloys can be tailored through the regulation of α/β phase stability by alloying elements. Previous studies have shown that alloying elements influence microstructural characteristics by modifying the volume fraction of the α and β phases. More importantly, alloying elements influence deformation behavior and strengthening mechanisms by altering the free-energy difference between the α and β phases, transformation kinetics, and the tendency for metastable phase formation [39,40]. Therefore, phase stability is not only the basis for titanium alloy classification, but also a key factor governing compositional design, microstructural evolution, and service performance. Traditionally, alloying elements are classified as α stabilizers, β stabilizers, or neutral elements according to their influence on the β-transus temperature. However, recent studies suggest that this classification does not fully capture the complex roles of alloying elements in titanium alloys. Their strengthening effects are also closely related to electronic structure, dislocation motion, and diffusion kinetics [41,42,43].

Among the α-stabilizing elements used in titanium alloys, Al is generally regarded as the most important substitutional alloying element. Previous studies have demonstrated that Al increases the β-transus temperature, stabilizes the α phase, and contributes to solid-solution strengthening [60,61]. From an atomistic perspective, first-principles calculations reported by Yu et al. [62] further revealed that increasing Al concentration decreases the generalized stacking fault energy (GSFE) for basal slip in α-Ti. In contrast, the GSFE associated with prismatic slip increases. This change in GSFE promotes basal slip activity and relatively suppresses prismatic slip, thereby modifying the relative activation tendency of different slip systems. As a result, multiple slip modes can participate more cooperatively in plastic deformation, which is beneficial for achieving an improved strength-ductility balance. Nevertheless, excessive Al addition may cause detrimental phase instability. When the Al content exceeds approximately 6.6 wt.%, the ordered α_2_-Ti_3_Al phase tends to precipitate. Although α_2_-Ti_3_Al can improve strength, its ordered structure markedly deteriorates ductility and fracture toughness [63,64,65].

**Figure 2:**
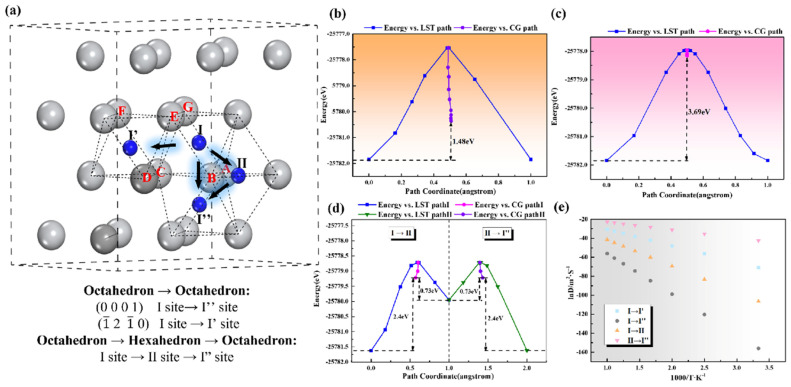
(**a**) Diffusion path of the N atom in α-Ti bulk; (**b**–**d**) energy profiles and diffusion energy barrier (eV) along different diffusion paths, with the lowest barrier of 1.48 eV highlighted; (**e**) calculated diffusion coefficients as a function of temperature for the three paths [70].

Interstitial elements, particularly oxygen (O) and nitrogen (N), are known to substantially strengthen titanium alloys via lattice distortion. Nevertheless, this strengthening is generally accompanied by a pronounced reduction in tensile ductility, a phenomenon commonly referred to as oxygen sensitivity [66,67]. A comprehensive summary by Luo et al. [68] further illustrates that increasing O content from 0.1 wt.% to 0.5 wt.% leads to a drastic decrease in room-temperature elongation of commercially pure titanium, from over 25% to below 5%. Nitrogen doping is an effective strategy for improving the surface and mechanical performance of titanium alloys. Nitrogen atoms preferentially occupy octahedral interstitial sites in the Ti lattice, where they induce lattice distortion, form interstitial solid solutions or nitrides, and hinder dislocation motion, thereby enhancing hardness, wear resistance, and corrosion resistance [69]. As illustrated in Figure 2, Liu et al. [70] showed that nitrogen diffusion in α-Ti exhibits the lowest energy barrier of 1.48 eV along the [$\overset{-}{1}$2$\overset{-}{1}$0] direction, suggesting a favorable migration pathway. Although Al reduces surface nitrogen adsorption, it lowers the subsurface diffusion barrier and thus promotes nitrided-layer growth. Moreover, nitrogen can increase the fraction of <c + a> dislocations, contributing to more uniform plastic deformation [71]. These findings indicate that nitrogen affects titanium alloys not only through interstitial strengthening but also by regulating diffusion behavior and slip activity.

In addition to α-stabilizing elements, β-stabilizing elements are another important class of alloying elements in titanium alloys, as they increase the proportion of the β phase and thereby improve processability and ductility. However, excessive β stabilization can compromise microstructural stability at elevated temperatures [72]. Although Sn and Zr have traditionally been considered neutral elements, recent studies indicate that Sn contributes to strengthening via lattice distortion, while Zr forms a homogeneous solid solution that maintains ductility. In multi-component β or metastable β alloys, Zr also significantly influences precipitation behavior and transformation pathways, suggesting that it should not be simply classified as neutral [73]. In engineering practice, the stability of the β phase is commonly assessed using the Mo equivalent (Mo_eq_). As Mo_eq_ increases, the α-phase fraction decreases, the β-phase fraction rises, and phase transformations shift from a martensitic to a diffusion-controlled regime. These changes are typically accompanied by reduced strength but improved ductility [74]. Because Mo_eq_ is an empirical parameter, it cannot fully capture the complex elemental interactions and metastable phase behavior in multi-component systems. Consequently, recent efforts increasingly integrate CALPHAD calculations with first-principles methods to enhance predictive accuracy [75,76].

In summary, alloying elements control titanium alloy performance not through a simple “stabilizer type → strength” relationship, but by jointly regulating phase stability, stacking fault energy, diffusion kinetics, and precipitation pathways. Beyond compositional effects, substrate integrity plays a critical role in aerospace titanium alloys. Inclusions-particularly hard alpha inclusions (TiN, TiC, or high-Al/O stabilized α regions)-and solute segregation can act as preferential crack initiation sites under cyclic or dwell fatigue loading, severely degrading low-cycle fatigue life and damage tolerance, especially in critical rotating components such as compressor discs and fan blades. Advanced detection techniques, including ultrasonic testing, X-ray computed tomography, and eddy current inspection, are employed for macroscopic defect screening, while scanning electron microscopy with energy-dispersive X-ray spectroscopy and electron probe microanalysis are used for detailed characterization of inclusion chemistry and morphology.

### 3.2. Important Microstructural Features

The macroscopic mechanical properties of titanium alloys are ultimately governed by how the microstructure regulates dislocation motion, deformation-mode selection, and strain partitioning. Advanced characterization techniques, including electron backscatter diffraction, transmission electron microscopy, and digital image correlation, have been extensively employed to reveal these governing relationships by providing direct observations of slip activity, strain localization, and crack initiation at the micro- and nanoscale. Therefore, rather than relying solely on empirical classifications of microstructural types, it is necessary to establish key microstructural descriptors that capture these regulatory effects. Such an approach is more suitable for developing a unified microstructure—property relationship framework. For titanium alloys, the most relevant descriptors include feature size, morphology, topology, crystallographic orientation, and interface structure [77,78].

Feature size mainly affects the mean free path of dislocations and the barrier effect of interfaces, thereby controlling strength and deformation uniformity. In general, microstructural refinement enhances strength. For example, Yao et al. [79] showed that reducing the hatch spacing from 90 μm to 30 μm refined the α′ martensite lath thickness and increased the yield strength from 981 MPa to 1213 MPa, following a Hall-Petch-type relationship. However, excessive refinement may limit slip redistribution among different slip systems and microstructural regions, increasing the risk of local strain concentration. Thus, the size effect is important not only for strengthening but also for maintaining local deformation stability during dwell fatigue and early damage.

**Figure 3:**
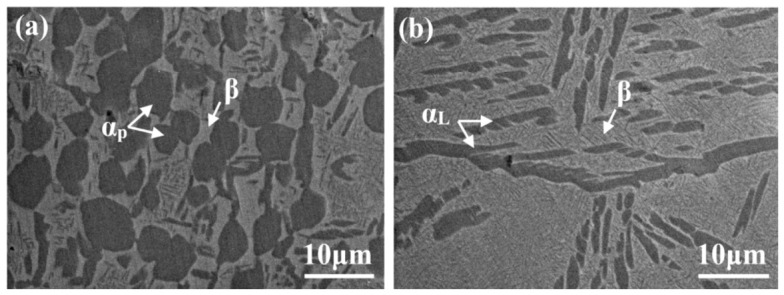
(**a**) bimodal microstructure; (**b**) lamellar microstructure [80].

Morphology and topology determine deformation-transfer pathways and strain distribution. Figure 3 shows typical bimodal and lamellar microstructures before deformation, illustrating the distinct morphological features discussed below [80]. Lamellar microstructures, characterized by strong interfacial constraints, can maintain high-temperature strength but may also promote localized deformation [81]. In contrast, equiaxed microstructures facilitate cooperative slip activation and strain dispersion, thereby improving ductility and fatigue resistance. Li et al. [82] found that the equiaxed microstructure in Ti-4Al-0.005B exhibited higher fracture elongation than the lamellar counterpart, mainly because easier slip transfer across grain boundaries promoted more uniform deformation.

Crystallographic orientation, especially texture and microtextured regions (MTRs), controls the activation and spatial distribution of deformation mechanisms. Strong textures enhance slip anisotropy and lead to preferential activation of specific slip systems. As reviewed by Kannan et al. [77], such textures in additively manufactured titanium alloys induce mechanical anisotropy and influence fatigue crack paths. These effects are closely related to MTR-induced strain incompatibility and stress concentration, which are critical factors in early fatigue crack initiation and propagation.

Interface and phase-boundary structures also play a dual role. They can impede dislocation motion and enhance strength, but they may also become preferential sites for stress concentration and crack initiation when dislocation transfer or strain accommodation is insufficient. Therefore, the effect of interfaces depends not merely on their presence, but on their ability to mediate dislocation transfer and local strain compatibility [83].

### 3.3. Microstructure Effects on Damage

In aerospace titanium alloys, microstructure serves as a key link between phase stability, dislocation behavior, and deformation-mechanism selection. By regulating dislocation transfer, local strain partitioning, and crack initiation or propagation paths, microstructural morphology largely determines the overall mechanical response. The overall mechanical response includes strength, ductility, fatigue resistance, fracture toughness, and high-temperature stability. Therefore, this section focuses on representative microstructural types. It discusses how they influence damage evolution by controlling these key deformation and fracture processes. Particular attention is also given to the service-condition dependence of these microstructure-mediated responses.

For α + β titanium alloys, lamellar, equiaxed, and bimodal microstructures are generally regarded as the three most representative microstructural architectures. Among them, lamellar microstructures, typically composed of elongated α laths embedded in a retained β matrix, have attracted considerable attention because of their superior fracture toughness and elevated-temperature stability. The orientation relationship between adjacent α and β phases strongly affects slip transfer, dislocation pile-up, and crack propagation. Their mechanical response is controlled not only by phase constitution, but also by the spatial arrangement of α/β lamellae and the crystallographic compatibility across α/β interfaces. Shi et al. [84] obtained a typical lamellar microstructure through triple heat treatment and showed that the alternating α/β lamellar architecture prolonged crack propagation paths and promoted crack deflection. This behavior suppressed long-range dislocation glide and delayed catastrophic crack coalescence. Similar effects have been reported in studies on high-temperature deformation and creep, where lamellar colonies enhanced crack-growth resistance and creep durability through interface-mediated deformation constraints [85,86,87,88,89,90]. These findings indicate that the strength-toughness synergy of lamellar microstructures is closely related to the ability of α/β interfaces to redistribute local stress and interrupt continuous slip transmission.

The mechanical performance of lamellar microstructures is further governed by geometric parameters, particularly α-lath thickness and colony size. Refining α laths increases the density of α/β interfaces, thereby strengthening the barrier effect against dislocation motion and contributing to higher strength and improved creep resistance. Gao et al. [81] performed hot compression tests on Ti-6Al-4V alloys with different α-lamellar thicknesses and reported that peak stress increased linearly with the reciprocal square root of α-lath thickness, indicating a Hall-Petch-type strengthening response. However, excessive lath refinement may intensify local strain concentration and reduce crack-tip plastic accommodation, thereby deteriorating fracture toughness and ductility reserve. This suggests that α-lath thickness alone cannot fully determine mechanical performance. Instead, the combined effects of colony morphology, crystallographic orientation, and interface characteristics must also be considered. In particular, the spatial orientation distribution and misorientation of α laths strongly affect load transfer between α and β phases, thereby regulating deformation compatibility and crack initiation or propagation. Recent studies further indicate that deformation heterogeneity in lamellar titanium alloys arises from the dynamic interaction among slip anisotropy, interface constraints, and local stress redistribution, rather than from isolated microstructural parameters alone [91]. Therefore, although lamellar microstructures offer advantages in strength and creep resistance, their performance optimization requires coordinated control of lath thickness, colony size, orientation relationship, and interfacial stability through integrated composition–microstructure design.

Compared with lamellar microstructures, equiaxed microstructures typically consist of primary equiaxed α grains with relatively random orientations and β phases distributed along grain boundaries [92]. Their main advantage lies in superior deformation compatibility. The primary equiaxed α phase facilitates the cooperative activation of multiple slip systems and promotes local strain accommodation [37,93]. For example, Jha et al. [93] performed Schmid factor analysis on deformed equiaxed Ti-6Al-4V microstructures and reported that the prismatic and pyramidal <a> and <c + a> slip systems exhibited significantly higher Schmid factors than the basal slip system, with *κ* values exceeding 0.4.

The bimodal microstructure consists of primary equiaxed α grains and transformed β regions containing fine secondary α laths. This architecture provides an effective strategy for balancing strength and ductility in α + β titanium alloys, as demonstrated by Dixit et al. [37] in Ti-6Al-4V. Unlike microstructures that rely primarily on slip coordination or crack deflection, the bimodal microstructure induces pronounced hetero-deformation strengthening. This strengthening originates from multi-scale heterogeneous interfaces among the primary α phase, secondary α phase, and β matrix. Dixit et al. [37] reported that dislocation multiplication, slip transfer across α/α boundaries, dislocation pile-up at α/β interfaces, and β-lath fracture collectively govern the deformation behavior in bimodal microstructures. These results indicate that the synergistic interaction of multi-scale interfaces is the key factor underlying the enhanced strength-ductility combination.

In addition to the morphology of the α phase, the volume fraction and spatial distribution of the β phase significantly influence the overall mechanical response. An appropriate amount of β phase enhances ductility and deformation compatibility while maintaining a certain level of strength. However, when the β phase content becomes excessively high, the yield strength may decrease [94]. In metastable β titanium alloys, the β phase can undergo stress-induced transformation to form α′ or α″ martensite during loading. This transformation provides an additional source of plasticity and improves the strength-ductility synergy. Chen et al. [95] demonstrated that stress-induced β → α″ martensitic transformation occurs in a metastable β-Ti-11Mo alloy during tensile deformation. The progressively formed α″ martensite plates act as effective barriers to dislocation motion, dynamically reducing the mean free path and promoting dislocation storage. This mechanism significantly enhances strain hardening, resulting in an excellent strength-ductility combination (yield ratio 0.63, total elongation 61%).

In multiphase titanium alloys, α/β interfaces, twin boundaries, and precipitate interfaces are key microstructural features that govern deformation behavior and damage evolution [78,96]. These interfaces can impede dislocation motion and contribute to strengthening; however, when dislocation transfer or strain accommodation is insufficient, they may also act as preferential sites for local stress concentration. Wan et al. [97] used molecular dynamics simulations of Ti-alloy bicrystals and showed that α/β interfaces can serve as preferential sites for dislocation nucleation while inducing stress concentrations in disordered interfacial regions. Their results further revealed that the dominant deformation mode, including prismatic slip, shear banding, basal slip, or pyramidal slip, is highly sensitive to the crystallographic orientation of the interface. This finding indicates that the mechanical role of interfaces is not fixed, but strongly depends on local crystallographic compatibility and deformation accommodation.

It is worth emphasizing that the regulation of damage evolution by typical microstructures is strongly dependent on service conditions. Under moderate- to high-temperature conditions, dislocation climb, interface migration, and microstructural coarsening gradually become dominant factors. Lamellar microstructures generally provide advantages in high-temperature strength and creep resistance by prolonging crack propagation paths and suppressing certain grain-boundary-related damage. However, long-term exposure may induce lath coarsening or reduce phase stability, thereby compromising high-temperature stability and damage tolerance [90]. Under cyclic loading, bimodal microstructures and relatively uniform equiaxed microstructures are more favorable for achieving homogeneous local strain distribution, which delays fatigue crack initiation [98]. In contrast, coarse lamellae or continuous grain-boundary α phases tend to promote strain localization and early crack formation.

For metastable β titanium alloys, dynamic deformation mechanisms, such as transformation-induced plasticity (TRIP) and twinning-induced plasticity (TWIP), play a crucial role in regulating fatigue damage evolution. Previous studies have shown that stress-induced α″ martensitic transformation and deformation twinning can be activated in metastable β titanium alloys, thereby improving strain-hardening capacity and plastic deformation compatibility [99]. However, most TRIP/TWIP-related studies have focused on monotonic deformation and tensile strain-hardening behavior, while the roles of crack-tip-induced twinning and martensitic transformation during cyclic loading remain insufficiently quantified. Jin et al. [100] reported that {332} <113> twins and α″ martensite can form near crack tips under cyclic loading. The synergistic interaction between these two deformation mechanisms promotes crack deflection and increases crack-path tortuosity, thereby enhancing fatigue crack growth resistance. These findings indicate that the microstructure of metastable β titanium alloys continuously evolves during cyclic loading. Therefore, fatigue damage in these alloys should be understood as a coupled process involving local phase stability, crack-tip stress concentration, and dynamic microstructural evolution.

**Figure 4:**
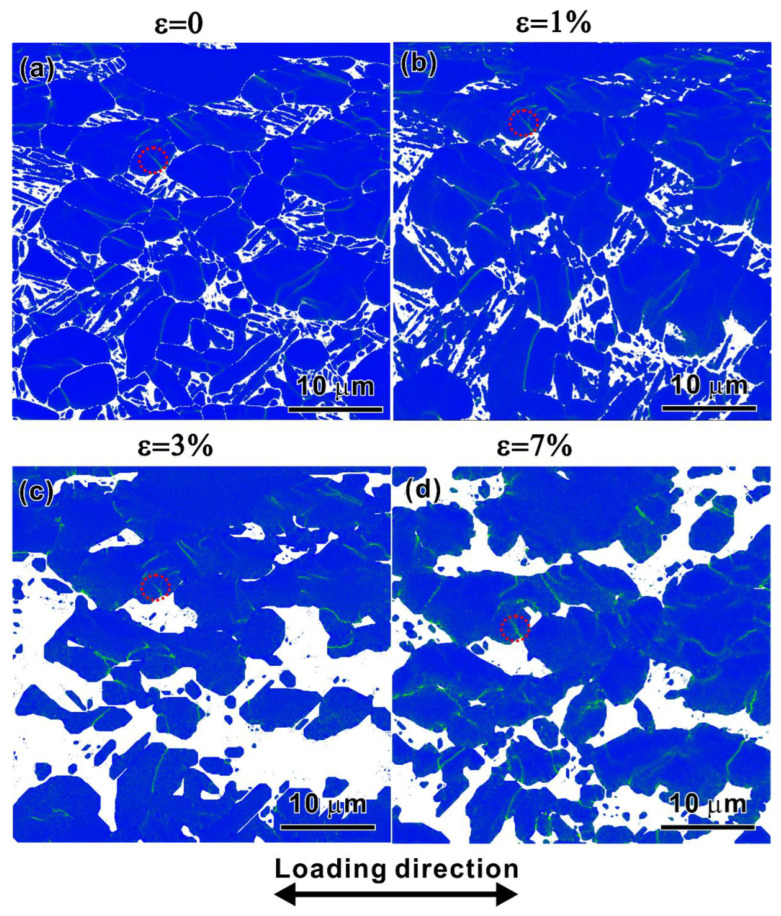
GND density maps obtained from EBSD data at true strains of (**a**) 0%, (**b**) 1%, (**c**) 3%, and (**d**) 7%, respectively [104].

From the perspective of damage initiation, differences among typical α + β titanium alloy microstructures mainly arise from their distinct regulation of dislocation transfer, local strain partitioning, and stress concentration sites. Lamellar microstructures generally consist of prior β grains containing α/β lath colonies that follow the Burgers orientation relationship. Within individual colonies, the relatively high slip geometric compatibility between α and β phases allows dislocations to transfer across multiple laths along continuous slip bands, contributing to crack deflection and high-temperature load-bearing capability. However, such long-range slip transfer may also promote local instability once dislocations are blocked by colony boundaries or grain-boundary α phases [101]. The resulting dislocation pile-up induces stress concentration and increases the probability of early crack nucleation. Zhou et al. [102] investigated damage initiation in lamellar microstructures through compression experiments combined with ex situ EBSD and KAM analysis. Their results revealed that cracks preferentially nucleate near colony boundaries with orientation discontinuities, even at relatively small macroscopic strains. High KAM values in these regions further indicate that localized strain accumulation is a key factor responsible for early cracking. Therefore, the limitation of lamellar microstructures is not restricted to the crack propagation stage; it is also closely associated with early damage nucleation at colony boundaries, grain-boundary α phases, and orientation-discontinuous regions. In contrast, equiaxed microstructures are generally considered to reduce damage initiation by enhancing local deformation compatibility. The intergranular β phase can buffer stress concentrations between adjacent primary α grains and facilitate slip transfer along α-β-α paths. This process promotes more homogeneous plastic deformation and reduces the likelihood of grain-boundary cracking. However, the improved deformation compatibility is often accompanied by reduced constraints on dislocation motion, thereby limiting further strengthening [103]. Bimodal microstructures offer a more balanced combination of strength and deformation accommodation because of the heterogeneous interfaces among primary α, secondary α, and the β matrix. Figure 4 provides direct experimental evidence from a bimodal Ti-6Al-4V alloy: as the tensile strain increases from 0% to 7%, the geometrically necessary dislocation (GND) density progressively increases and localizes near α/β interfaces and within primary α grains [104]. Strain gradients and geometrically necessary dislocation structures generated near multiscale α/β interfaces can enhance work-hardening capacity, delay local plastic instability, and suppress premature crack initiation. Accordingly, bimodal microstructures are often regarded as more favorable than fully lamellar or fully equiaxed microstructures for achieving a balance among strength, ductility, and damage tolerance [37]. Nevertheless, it remains difficult to quantify when beneficial strain partitioning evolves into harmful strain localization in bimodal microstructures, particularly under complex loading conditions. Future studies should therefore establish quantitative links among local orientation mismatch, GND accumulation, crack-nucleation sites, and macroscopic fatigue resistance.

In addition to the internal microstructural features discussed above, compressive residual stresses introduced near the surface through mechanical surface treatments, such as shot peening, laser shock peening, and deep rolling, play a critical role in retarding fatigue damage initiation and early propagation in titanium alloy components [105]. These compressive stress layers effectively close fatigue cracks and reduce the effective driving force for crack growth, thereby substantially improving high-cycle fatigue and fretting fatigue resistance. The beneficial effects arise from the superposition of residual compressive stresses with service-induced tensile stresses, which lowers the local mean stress and delays crack nucleation at surface imperfections or slip bands [106]. However, the stability of these compressive stress layers under cyclic loading at elevated temperatures or in aggressive environments requires careful evaluation, as stress relaxation, thermal recovery, or surface degradation can diminish their protective effect over extended service lives.

In summary, the three microstructural architectures regulate damage evolution through fundamentally distinct pathways, each carrying intrinsic trade-offs. Lamellar microstructures provide superior high-temperature crack deflection and creep resistance but suffer from local strain concentration at colony boundaries, which promotes early damage nucleation and reduces room-temperature ductility. Equiaxed microstructures achieve homogeneous strain partitioning and excellent fatigue resistance through cooperative slip, yet their weak dislocation constraints limit strength. Bimodal microstructures balance these extremes via multi-scale heterogeneous interfaces that generate strain gradients and delay crack initiation, though their performance window remains sensitive to phase proportions. No single microstructure maximizes all properties simultaneously; their superiority is service-condition dependent. High-temperature components favor lamellar structures, fatigue-critical parts benefit from equiaxed or bimodal designs, and comprehensive scenarios require tailored bimodal architectures.

### 3.4. Microstructure Selection for Service

The previous section demonstrated that lamellar, equiaxed, bimodal, and metastable β microstructures govern damage evolution through distinct mechanisms. Lamellar microstructures exhibit superior creep resistance and enhanced crack-deflection capability; however, they are susceptible to localized strain concentrations at colony boundaries. Equiaxed microstructures promote homogeneous strain partitioning and excellent fatigue resistance; however, limited dislocation constraint reduces their strength. Bimodal microstructures achieve a favorable balance between strength and ductility through multiscale heterogeneous interfaces; however, their performance is highly sensitive to phase fractions. Metastable β microstructures provide additional strain-hardening capacity through TRIP/TWIP mechanisms; nevertheless, their damage tolerance under cyclic loading conditions requires careful evaluation. Since no single microstructure can simultaneously optimize all performance attributes, microstructural selection should be guided by the prevailing service conditions. Table 2 summarizes the recommended microstructural selection strategy for different service requirements.

## 4. Competing Deformation and Phase Transformation Mechanisms

The mechanical properties of titanium alloys are governed not only by phase composition but also by phase-specific deformation mechanisms and their interactions. In α + β and metastable β titanium alloys, multiple deformation modes, including slip, twinning, and stress-induced phase transformation, may coexist or compete depending on stress state and phase stability. Therefore, understanding the coupling among these mechanisms is essential for establishing robust composition–microstructure–property relationships. α-Ti possesses an HCP structure. At room temperature, plastic deformation in α-Ti is mainly accommodated by prismatic <a> slip, basal <a> slip, and pyramidal <c + a> slip [101]. Large differences in the critical resolved shear stress among these slip systems result in pronounced mechanical anisotropy. Prismatic <a> slip is readily activated, whereas pyramidal <c + a> slip requires a higher activation stress. Consequently, local strain concentration can readily develop at low temperatures or under complex stress states [107]. The substitutional element Al can modulate the generalized stacking fault energy, alter the competition among slip systems, and improve plastic deformation uniformity. Interstitial elements such as O and N can strengthen the α phase. However, they may also suppress cross-slip and promote strain localization. The synergistic effect of O and Al can partially restore slip dispersion. This effect may help rebalance strengthening and plasticity. Meanwhile, N may act as an additional alloying variable for regulating slip pathways [46,108,109,110]. β-Ti possesses a body-centered cubic (BCC) structure. In principle, this structure provides more available slip systems and therefore a higher potential for ductile deformation. However, its actual deformation behavior is strongly governed by β-phase stability [39]. In stable β alloys, plastic deformation is primarily controlled by dislocation slip. Strengthening in these alloys generally arises from solid-solution strengthening, precipitation strengthening, and dislocation strengthening. In contrast, metastable β alloys can activate stress-induced deformation mechanisms, including transformation-induced plasticity and twinning-induced plasticity [39,111,112]. Fu et al. [99] systematically summarized how β-phase stability governs the selection of deformation mechanisms in metastable β titanium alloys. Their analysis suggests that, with increasing β-phase stability, the dominant deformation mechanism tends to evolve from transformation-induced plasticity to combined transformation- and twinning-induced plasticity, and finally to dislocation slip. This mechanism transition strongly affects strain-hardening behavior and consequently influences the balance between strength and ductility.

### 4.1. Slip and Deformation Modes in α Alloys

The plastic deformation of α and near-α titanium alloys is governed by the HCP crystal structure of the α phase. The limited number of independently activatable slip systems results in pronounced crystallographic anisotropy. This anisotropy causes individual grains to respond differently to external loading. It also affects local strain distribution, fatigue-damage susceptibility, and crack initiation [32,33,34,113].

In real microstructures, prismatic <a> slip is often considered the easiest slip mode to activate. However, slip-system activation is often controlled more strongly by grain orientation, interphase constraints, and local stress states than by the nominal critical resolved shear stress. Liu et al. [114] investigated a near-α titanium alloy under low-stress four-point bending fatigue at 90% of the proof stress. Although the CRSS values for basal and prismatic <a> slip are comparable, approximately 325 and 355 MPa, respectively, basal slip dominated the early stage of plastic deformation. Specifically, among the slip-active primary α grains, 55 exhibited basal slip, whereas only one exhibited prismatic slip. Their inverse pole figure analysis showed that grains undergoing basal slip were consistently located near the [0001] pole, indicating that their c-axes were nearly parallel to the loading direction. Because the stiffness along the c-axis is approximately 50% higher than that along the <a> direction, these grains experience higher local stresses and therefore activate basal slip early, even when their Schmid factors are not maximal. Moreover, their 3D EBSD reconstruction revealed that the grain containing a transgranular crack was tightly surrounded by hard secondary α laths. This finding suggests that adjacent hard phases can strongly constrain slip-band propagation. Overall, these results indicate that crystallographic orientation, elastic-anisotropy-induced local stress concentrations, and phase-boundary constraints can outweigh nominal CRSS in determining slip-system selection in multiphase titanium alloys.

**Figure 5:**
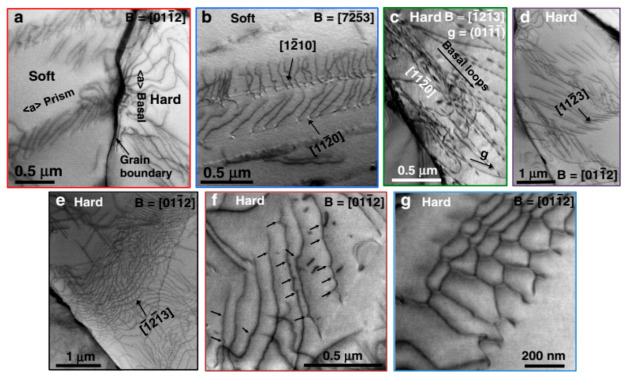
Dislocation structures in soft/hard grain pair: (**a**) basal dislocation nucleation; (**b**) prism pile-ups; (**c**) basal loops; (**d**,**e**) c + a dislocations; (**f**) cross-slip; (**g**) c + a network [116].

Although basal <a> slip contributes only modestly to macroscopic yielding, it can readily induce strain concentration in locally constrained grains and thereby provide a critical pathway for intergranular crack initiation [115]. Figure 5 illustrates the grain-boundary dislocation interaction observed by Xu et al. [116] using transmission electron microscopy. Prismatic dislocation pile-ups generated in a soft grain impinge on an adjacent hard grain and nucleate basal dislocations within the hard grain at the grain boundary. Such dislocation accumulation produces severe local stress concentration at the boundary, which is a preferred site for crack initiation. Joseph et al. [117] further reported that when slip is blocked at a grain boundary, intense dislocation activity and pile-ups occur, rendering the boundary highly susceptible to cracking. Jia et al. [118] provided complementary macroscopic evidence that, under transverse loading, fatigue crack initiation facets are flat and featureless, characteristic of intergranular quasi-cleavage fracture. Collectively, these findings establish that local crystallographic constraints—particularly hard-oriented α grains with their c-axes nearly parallel to the loading direction—amplify basal-slip-induced strain concentration at grain boundaries, directly explaining the susceptibility to intergranular crack initiation in textured near-α and α + β titanium alloys.

Microstructural morphology and microtexture can further amplify slip anisotropy. Lamellar α-lath colonies can promote long-range slip localization. In bimodal microstructures, the mechanical mismatch between primary α and transformed β regions plays a key role in stress redistribution. Chong et al. [119] combined nanoindentation with micro-digital image correlation (micro-DIC) to examine hardness disparity and plastic strain partitioning between primary α (αp) and transformed β (βtrans) regions in a bimodal Ti-6Al-4V alloy. They observed that when the nanohardness of αp markedly exceeded that of βtrans by 0.4–0.8 GPa, plastic strain initially localized in the softer βtrans regions during the early stage of tensile deformation, at approximately 1.0% plastic strain. With increasing strain, plastic deformation gradually transferred from βtrans regions to adjacent αp grains across the αp/βtrans interfaces, generating a pronounced strain gradient. These results indicate that the mechanical mismatch between αp and βtrans governs the stress-redistribution pathway in bimodal microstructures. In addition, grain clusters in microtextured regions can form soft/hard partitions and intensify load transfer [91]. Therefore, the deformation behavior of α and near-α titanium alloys should be understood within an integrated framework that links deformation mode, local strain accommodation, and damage initiation. Prismatic <a> slip generally promotes relatively homogeneous plastic flow. Basal <a> slip, although not dominant in macroscopic yielding, plays a key role in early local damage initiation [101,114]. By contrast, <c + a> slip and twinning can alleviate strain mismatch and delay damage accumulation. Together, these mechanisms provide a basis for synergistically optimizing deformation modes to balance plastic accommodation and damage resistance.

### 4.2. Solute Effects on Dislocation Motion

Solute elements play a critical role in regulating dislocation behavior in near-α titanium alloys. Their influence extends beyond conventional solid-solution strengthening and becomes an important factor governing deformation-mode selection and damage-evolution pathways. First-principles calculations have shown that alloying elements and interstitial atoms can influence the macroscopic mechanical response by modulating dislocation-core structure, slip-plane selectivity, and competition among different slip systems [120].

**Figure 6:**
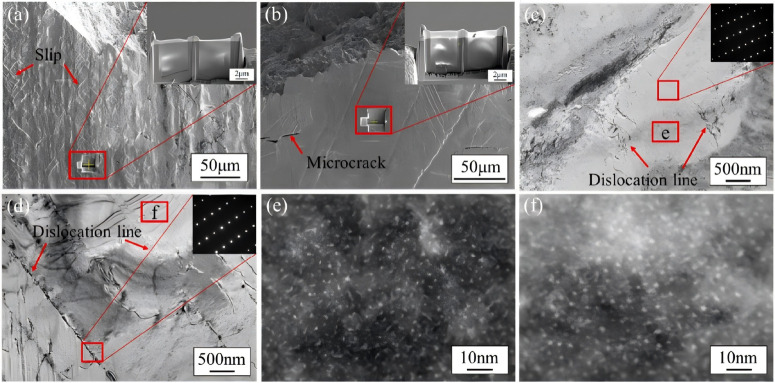
(**a**,**b**) FIB cutting positions near the fracture surface of TC4 alloy with low (PO20) and high (PO60) oxygen concentration alpha-cases, respectively; (**c**,**d**) corresponding TEM bright-field images showing dislocation morphologies: wavy slip with curved and entangled dislocations in PO20 (**c**) and planar slip with straight parallel dislocations in PO60 (**d**); (**e**,**f**) HAADF-STEM images revealing fine α_2_ (Ti_3_Al) precipitates (white particles) in both conditions [122].

In pure α-Ti, < a>-type screw dislocations can undergo cross-slip, producing tortuous dislocation paths and dispersing plasticity across multiple slip planes [121]. In contrast, in near-α titanium alloys containing solutes such as Al and O, dislocation motion tends to become more confined. This confinement promotes characteristic planar slip, mainly attributed to elastic interactions between solutes and dislocations, together with solute-induced modulation of dislocation-core electronic structure. The transition from wavy to planar slip with increasing solute concentration has been directly observed by transmission electron microscopy. As shown in Figure 6c, when the oxygen concentration in the alpha-case of a TC4 alloy is low, dislocations exhibit a curved and entangled morphology, characteristic of wavy slip that permits cross-slip and multislip deformation. In contrast, at high oxygen concentration, dislocations become straight and parallel (Figure 6d), indicating a fully planar slip mode [122]. Notably, nanoscale α_2_ precipitates are present in both conditions (Figure 6e,f), yet planar slip only emerges at high oxygen content. This suggests that dissolved oxygen, rather than α_2_ alone, is the dominant factor forcing dislocation motion onto fewer slip planes. The accompanying lattice expansion along the c-axis further confirms that interstitial oxygen modifies the dislocation core structure and restricts slip planarity [122]. In addition to the direct effect of oxygen solutes, the formation of nanoscale α_2_ (Ti_3_Al) ordered phases further promotes planar slip. These precipitates impose a high critical resolved shear stress for the first dislocation cutting event, but the resistance to subsequent dislocations moving on the same slip plane is substantially reduced, thereby promoting dislocation pairing and planar slip. Further evidence shows that α_2_ precipitates formed along α/β interfaces act as effective obstacles to dislocation motion [123], leading to dislocation pile-ups and bundles at these interfaces. Consequently, interfacial constraints induce dislocation bundling within planar slip bands. Thus, solute regulation not only modifies solid-solution strengthening but also restricts slip propagation and promotes slip planarity through the formation of α_2_ ordered phases, enhancing local slip-mode constraint.

Solute-induced planar slip can modify the relative activation barriers of different slip systems. Within α-lath colony structures, basal <a> slip may dominate plastic deformation under specific conditions. Solutes such as Al reduce the differences in critical resolved shear stress (CRSS) among various slip systems. This promotes redistribution of dislocations across multiple slip planes, giving rise to a complex mixed planar-wavy slip morphology [124]. Under low strain-rate or stress-hold conditions, basal <a> slip is preferentially activated. This preferential activation generates local stress concentrations at grain boundaries or α/β interfaces. These local stress concentrations constitute a key microscopic mechanism for dwell-fatigue crack initiation. Transmission electron microscopy and 3D EBSD observations indicate that transgranular crack facets form parallel to basal slip traces, with the crack plane deviating only ~6° from the basal plane, confirming that basal slip directly governs crack initiation [114]. When the interfacial geometric compatibility factor (m′) is low, dislocations struggle to transfer across the interface. This leads to local strain accumulation and intensified stress. Conversely, when the interface is favorable for slip transfer, local strain can be effectively dispersed. This effectively delays damage accumulation.

In summary, solute-mediated regulation of dislocation behavior in near-α titanium alloys reconfigures microscopic plasticity pathways by altering dislocation core structures and the slip-energy landscape. The significance of this deformation-path selection effect extends beyond conventional strengthening. It is directly associated with strain localization, damage nucleation, and service reliability. Future studies should integrate atomic-scale simulations with high-resolution experimental characterization. Such approaches are needed to quantify the dynamic effects of solutes on the CRSS of individual slip systems and on dislocation migration. Cross-scale physics-based models should also be established. These models would provide a theoretical basis for designing near-α titanium alloys with enhanced damage tolerance.

### 4.3. Twins and Deformation

**Figure 7:**
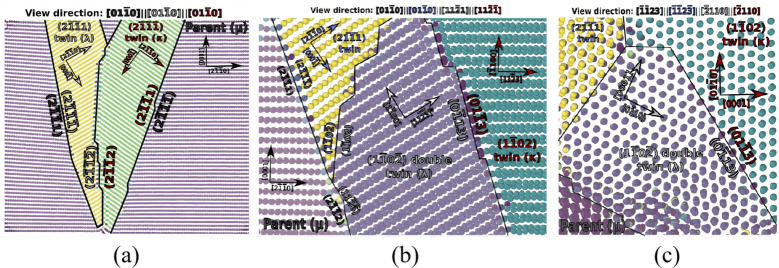
(**a**) Twin–twin interaction between co-zonal (2$\bar{111}$) (yellow) and (2$\bar{111}$) (green) twins. The twin–twin interface is a (2$\bar{11}$2) twin boundary with limited mobility; steps can nucleate and move but triple points cannot move conservatively. (**b**,**c**) Interactions between a (2$\bar{111}$)–($\bar{11}$0$\bar{2}$) double twin and a (1$\bar{1}$02) twin. All four lattice orientations are visible. (**b**) shows the shear plane direction for the initial (2$\bar{111}$) twin; (**c**) shows the (0$\bar{11}$3) twin plane interface viewed from the shear plane direction [131].

In near-α and α + β titanium alloys, twinning not only provides additional strain along the <c> axis, but also participates in local plastic accommodation through lattice reorientation and interface migration [120,125,126]. Gong et al. [120] demonstrated that <a> dislocations can be absorbed and transmuted at twin boundaries, directly affecting dislocation transfer. Zhou et al. [125] further revealed that prismatic dislocations undergo sequential transmutation during twin–slip interaction, altering deformation paths. Qian et al. [127] and Zhao et al. [128] showed that twinning can induce lattice reorientation and dynamically refine grains, thereby influencing microstructural evolution and work hardening. Twin–twin interactions, as studied by Huang et al. [129] and Li et al. [130], also contribute to strain accommodation and boundary migration. Figure 7a shows an atomistic simulation of two co-zonal {11$\overset{-}{2}$1} twin variants in titanium. Their interaction creates a new {11$\overset{-}{2}$2} twin boundary at the intersection [131], confirming that twin–twin interactions generate new interfaces and locally reorient the lattice. Moreover, Zheng et al. [126] observed that {11$\overset{-}{2}$1} twins can stimulate secondary {10$\overset{-}{1}$2} twins in the β phase, illustrating how twinning participates in local plasticity through stress-induced reorientation. Various twinning systems in hcp-Ti are summarized in Table 3. Therefore, the role of twinning should not be limited to compensating for insufficient slip. It should also be understood in terms of its effects on dislocation transfer, deformation paths, and microstructural evolution.

Slip-twin interactions form the fundamental basis for understanding the role of twinning. Research has demonstrated that twin boundaries do not act as absolute obstacles to dislocation motion. Instead, dislocations can dissociate, transfer, or undergo slip plane conversion at the interface. Chen et al. [132] reported that prismatic dislocations impinging on a {10$\overset{-}{1}$1} twin boundary are transmuted into a growing internal {11$\overset{-}{2}$1} twin, which later transforms back into prismatic dislocations on the opposite side. Further analysis reveals that, in certain cases, the Burgers vector remains unchanged while the slip plane is altered, indicating that the process involves local crystallographic rearrangement rather than simple geometric continuation. Moreover, the transfer of slip to twins is highly sensitive to the local grain boundary morphology. Wang et al. [133] observed co-located prismatic slip bands and {10$\overset{-}{1}$2} tensile twins in commercially pure titanium, suggesting that slip can stimulate twin nucleation across grain boundaries. However, geometric factors alone, such as the m′ parameter, are insufficient to predict such transmissions [134]. Han and Crimp [135] further demonstrated that the same grain boundary can exhibit different slip transmission behaviors at different depths solely due to variations in the boundary orientation relative to the incoming slip bands, highlighting the critical role of local boundary morphology. Curved boundaries or pre-existing shear bands intensify stress concentration, as shown by the enhanced local stress fields at slip-band tips in bicrystal simulations [133]. Li and Chew [136] also revealed through molecular dynamics that grain boundary shear tractions can either assist or hinder dislocation emission, depending on their alignment with the applied stress. This increases the likelihood of slip-twin transformation. Consequently, the activation and propagation of twinning are not isolated processes. They are closely coupled with the local slip field and interfacial conditions.

Beyond interfacial interactions, twin variant selection critically influences the overall contribution of twinning to deformation. Studies indicate that a substantial number of low Schmid factor or anomalous twin variants can emerge in near-α titanium alloys under room-temperature and cryogenic conditions. These observations suggest that variant activation is governed not only by external loading, but also by local constraints and strain compatibility. In pure titanium, low-SF twins are frequently observed and linked to multiple variants within the same grain [137,138]. Zhou et al. [30] further showed that cryogenic rolling promotes continuous high-order sequential twinning (CTW→ETW→CTW→ETW). Each twinning step reorients the lattice, redefining conditions for subsequent deformation. Thus, twinning should not be regarded as a single localized response, but rather as a dynamic process that persistently influences microstructural refinement and strain hardening.

Twinning and its reverse process significantly influence path-dependent plastic responses. Existing studies have shown that {10$\overset{-}{1}$2} tensile twins, {11$\overset{-}{2}$2} compressive twins, and detwinning behavior are important sources of tension-compression asymmetry and the Bauschinger effect in hexagonal close-packed (HCP) titanium alloys. Under reverse loading or cyclic deformation conditions, detwinning not only alters the local orientation distribution, but also reshapes the load-bearing capacity for subsequent slip. As a result, strain distribution and damage accumulation are continuously affected. Therefore, under service conditions, twinning-detwinning behavior can serve as a key internal variable for describing the effect of loading history.

Based on the above discussion, the role of twinning in near-α and α + β titanium alloys extends beyond providing additional strain. A more complex example is presented in Figure 7b,c, where a {11$\overset{-}{2}$1}–{10$\overset{-}{1}$2} double twin interacts with a {10$\overset{-}{1}$2} twin in the parent grain. This compound reaction produces a new {01$\overset{-}{1}$3} twin boundary. The images reveal all four lattice orientations involved, and the boundary is viewed along two different directions (shear plane direction of the initial {11$\bar{2}$1} twin in (b), and the {01$\overset{-}{1}$3} twin plane interface in (c)) [131]. Such hierarchical twin–twin interactions demonstrate that twinning can continuously reshape the deformation landscape through multi-order reactions, creating new mobile interfaces and altering local crystallography. Through slip–twin interactions, variant selection, detwinning, and transformation mechanisms, twinning continuously influences the local stress state, work hardening, and microstructural evolution path. Therefore, when the deformation behavior of such alloys is analyzed, twinning should be regarded as a mechanism comparable to slip and phase transformation, rather than merely as a secondary accommodation process.

### 4.4. ω Phase in β Alloys

In metastable β titanium alloys, the ω phase is not merely an incidental microstructural byproduct formed during phase transformation. Instead, it acts as a key microstructural unit that directly influences deformation path selection. Its core role does not lie in simply altering the phase composition. Instead, it lies in reconstructing the competitive relationships among slip, twinning, and stress-induced phase transformation through the modulation of dislocation motion and the local stress state [99,139,140].

Depending on the formation pathway, the ω phase is typically classified into athermal ω and isothermal ω. Although these phases form under different conditions, both can significantly increase the resistance to dislocation motion within the β matrix. Finely dispersed ω precipitates act as high-density obstacles and enhance yield strength. However, they also promote slip planarity and deformation localization. As a result, plastic deformation is accommodated by only a few slip channels. Consequently, the strengthening effect of the ω phase is generally accompanied by reduced deformation compatibility. More importantly, the ω phase alters the relative priority of different deformation mechanisms. At low ω phase contents, metastable β alloys predominantly exhibit dislocation slip, which may be accompanied by TRIP or TWIP. However, when the ω phase precipitates in a dispersed manner, both dislocation slip and stress-induced phase transformation are suppressed. Local stress accumulation intensifies, and the material transitions toward a more localized plasticity mode [35,141].

This characteristic is typically demonstrated in the Ti-25Nb-0.7Ta-2Zr alloy. Lai et al. [35] subjected the solution-treated alloy to water quenching (ω-free) and furnace cooling, which produced a high density of nanoscale ω particles. The water-quenched alloy sequentially activated stress-induced β→α″ martensitic transformation, {332}β twinning and dislocation slip, giving rise to combined TRIP/TWIP effects and pronounced work hardening [142,143]. In contrast, the ω particles in the furnace-cooled alloy fully suppressed martensitic transformation and twinning, confining plasticity to ω-depleted dislocation channels and resulting in much lower work hardening [144]. Consequently, the yield strength increased from 350 MPa to 490 MPa, but uniform elongation dropped from 25% to 13%. These results demonstrate that the ω phase does not simply strengthen a single mechanism; rather, it globally alters the competitive balance among deformation mechanisms. By raising the critical shear stress and blocking the long-range propagation of martensite and twins [145,146], the ω phase forces plastic deformation to become highly localized within a few channels, sacrificing work hardening capacity and uniform elongation.

In essence, the role of the ω phase arises from its ability to impede the critical shear path required for the β→α″ transformation. As a result, the propagation capability of the stress-induced phase transformation is weakened. Consequently, the ω phase not only restricts dislocation motion, but also reduces the contribution of the TRIP mechanism to plastic accommodation [35,147]. This effect is particularly critical for metastable β titanium alloys that rely on the synergistic deformation of multiple mechanisms. However, the influence of the ω phase is not always equivalent to embrittlement. Instead, it depends significantly on the size and distribution state of the ω phase [141,148]. An appropriate amount of fine, uniformly dispersed isothermal ω phase can achieve a favorable strength-ductility balance in certain alloy systems. For example, in a Ti-5Mo-3Cr-1Zr alloy, Abro et al. [149] showed that fine ω precipitates formed during short-term low-temperature aging do not act as static obstacles; instead, they couple with twins, dislocation channels and α″ martensite evolution during deformation, thereby preserving ductility. In contrast, when ω coarsens, strength continues to increase but ductility drops sharply, exhibiting a typical strengthening-embrittlement transition [150]. Moreover, ω coarsening and local inhomogeneities at ω/β interfaces exacerbate stress concentration and increase the propensity for crack initiation [150].

### 4.5. Competing Deformation and Transformation

The plastic behavior of titanium alloys is governed by the dynamic interplay of multiple deformation mechanisms. In α and near-α alloys, plastic deformation primarily involves dislocation slip and mechanical twinning. In metastable β alloys, plasticity additionally involves stress-induced phase transformations and ω-phase evolution.

In α and near-α alloys, dislocation slip accommodates the majority of plastic deformation, while twinning is activated when local strain cannot be sufficiently accommodated. When dislocation slip is impeded by crystallographic constraints, solute atoms, or interfacial barriers, twinning is initiated to relieve strain mismatch. Thus, slip and twinning act complementarily, depending on the local microstructure and applied stress state.

In metastable β alloys, TRIP (transformation-induced plasticity), TWIP (twinning-induced plasticity), and ω-phase evolution contribute to plastic deformation in conjunction with dislocation slip. The ω phase increases slip resistance, alters the conditions for phase transformations, and modifies the interplay among deformation mechanisms. Consequently, the dominant deformation mechanisms evolve dynamically with spatial position and deformation stage.

The competition among deformation mechanisms strongly depends on the microstructure. Phase composition, interfacial structures, crystallographic texture, and local constraints govern the activation sequence of deformation mechanisms. In regions with high local constraints, accommodation mechanisms such as twinning or phase transformation are preferentially activated, whereas in regions where slip is unhindered, dislocation slip predominates. Thus, the competition among deformation mechanisms is a multiscale process regulated by crystallographic constraints, solute effects, and microstructural characteristics. From an aerospace engineering perspective, the practical significance of understanding these competing mechanisms lies in their direct influence on component-level performance indicators such as high-cycle fatigue life, dwell fatigue susceptibility, and creep-rupture resistance. Tailoring the dominant deformation mode-whether through alloy composition, thermomechanical processing, or surface treatment-enables the design of titanium alloys with optimized combinations of strength, ductility, and damage tolerance for specific flight-critical applications.

## 5. Conclusions and Outlook

This review has systematically examined the multiple deformation mechanisms in aerospace titanium alloys and their dependence on microstructural features, with particular emphasis on the competition and synergy among dislocation slip, deformation twinning, and stress-induced phase transformations. The regulatory roles of microstructure and processing in determining macroscopic properties are also critically discussed. The key findings are summarized as follows:

(1) Alloying elements and phase stability. Alloying elements regulate the mechanical performance of titanium alloys through multiple pathways beyond simple phase stabilization. Al modulates generalized stacking fault energy to promote multi-slip activation, while excessive Al addition induces α_2_-Ti_3_Al ordering that compromises ductility. Interstitial O and N provide substantial strengthening through lattice distortion but promote planar slip and strain localization. β stabilizers such as Mo, V, and Cr control β-phase retention and transformation kinetics. The Mo equivalent remains a useful empirical guide, yet its limitations highlight the need for CALPHAD and first-principles integration.
(2) Microstructural control of mechanical performance. Typical microstructural morphologies (lamellar, equiaxed, and bimodal), together with crystallographic texture, interface characteristics, and feature size, govern the strength, ductility, fatigue resistance, and damage tolerance of titanium alloys. Lamellar microstructures provide superior fracture toughness and creep resistance through crack deflection, equiaxed microstructures facilitate homogeneous strain partitioning and fatigue resistance, and bimodal microstructures achieve a balanced strength-ductility combination through multiscale heterogeneous interfaces. These effects are realized through modulation of dislocation motion pathways, activation sequence of competing mechanisms, and spatial distribution of localized strain. The optimal microstructure is service-condition dependent.
(3) Competitive and synergistic switching of deformation mechanisms. The strength, ductility, and fatigue performance of titanium alloys are jointly determined by the relative activities of slip, twinning, and phase transformation. Variations in local strain partitioning and deformation compatibility originate from the competitive and synergistic switching among these mechanisms under specific local constraints and stress states. In metastable β systems, ω-related processes (including isothermal ω and stress-induced ω) fundamentally reconfigure the contribution ratios of slip, twinning, and stress-induced phase transformations (e.g., β→α’ or β→α’’). Consequently, work hardening capacity and plastic uniformity are substantially altered, shifting deformation between localized and homogeneous modes depending on the prevailing ω-mediated competition path.
(4) Key challenges and identified research priorities. This review has identified several critical challenges that remain unresolved, particularly the quantitative characterization of multi-mechanism coupling and the role of local heterogeneity in dictating deformation-path selection. In addition, the need for predictive approaches capable of linking composition, processing, and microstructure to service performance is emphasized as a key direction for advancing titanium alloy design.

Several critical issues remain unresolved and warrant further investigation.

(1) A unified predictive framework needs to be established by integrating high-resolution in situ characterization—including in situ transmission electron microscopy, atom probe tomography, and synchrotron X-ray diffraction—with multi-scale modeling and first-principles calculations to clarify solute–dislocation interactions, the pathways of stress-induced phase transformations, and the detection of substrate defects such as inclusions and segregation at the micro- and nanoscale.
(2) AI-assisted and data-driven methods should be introduced to build quantitative composition–microstructure–property mappings, moving beyond empirical trial-and-error toward more efficient alloy design.

Ultimately, these efforts will support the rational design of next-generation aerospace titanium alloys with high specific strength, excellent microstructural stability, and superior fatigue resistance.

### Limitations of This Review

It should be acknowledged that this review has several limitations:

(1) The discussion primarily focuses on room-temperature to intermediate-temperature deformation behavior, with limited coverage of high-temperature creep and oxidation.
(2) Quantitative structure–property relationships and predictive modeling approaches are only briefly addressed.
(3) The unique microstructural features and deformation behaviors arising from additive manufacturing routes are not comprehensively covered.

These limitations are acknowledged and point toward promising directions for future research.

## Figures and Tables

**Table 1:** The properties of typical titanium alloys (UTS = Ultimate Tensile Strength, YS = Yield Strength, LPBF = Laser Powder Bed Fusion, AC = Air Cool, CR = Cold Rolled, WQ = Water Quench, FC = furnace cooling, PM = Powder Metallurgy).

Alloy	Type	Strength (MPa)	Hardness	Elongation (%)	Young’s Modulus (GPa)	Manufacturing/Heat Treatment
Ti-5Al-2.5Sn [44]	α	≈1500 (UTS) ≈1400 (YS)	308.5 HV [HV0.5]	12	-	LPBF
CP-Ti [45]	α	466.3 (YS)	-	-	100.5	CR
Ti-8Al-1Mo-1V [46]	Near-α	≈1025 (UTS)) ≈ 975 (YS)	-	≈20	-	1010 °C/1 h, WQ + 580 °C/8 h, AC
Ti-6Al-2Sn-4Zr-2Mo [47]	Near-α	1120 (UTS) 880 (YS)	329 ± 13 HV [HV0.3]	19.4	-	Forging 955 °C/1 h, AC
Ti-5.8Al-4.8Sn-3.5Zr-1.0Mo-0.35Si-0.85Nd-0.1C [48]	Near-α	1160–1174 (UTS) 1015–1025 (YS)	-	9.4–10.6	-	Hotextrusion
Ti-6Al-2.8Sn-4Zr-0.5Mo-0.4Si-0.1Y [48]	Near-α	995–1000 (UTS) 915–925 (YS)	-	11.5–15	-	Forged aged solution
Ti-6Al-2.7Sn-4Zr-0.4Mo-0.4Si [48]	Near-α	1010–1050 (UTS) 900–950 (YS)	-	-	112	As cast
Ti-6Al-4V [49]	α + β	1126 (UTS) 1020 (YS)	363 ± 2 HV [HV1]	10.9	-	Cryorolling + Vacuum short annealing 700 °C/5 min, FC
Ti-6Al-7Nb [50]	α + β	≈900 (UTS)	280–300 HV [HV30]	≈3	-	PM + uniaxial cold pressing 1250 °C/120 min, 5 °C/min heat/cool
Ti-6Al-6V-2Sn [51]	α + β	1509 (UTS) 1437 (YS)	-	10.5	-	Forging and rolling 900 °C/1 h WQ + 520 °C/4 h AC
Ti-6Al-2Sn-4Zr-6Mo [52]	α + β	1209 (UTS) 1068 (YS)	396 HV [HV0.5]	12	119	Beta forging + sub-β solution + ageing 593 °C/8 h
Ti-6Al-2Sn-2Zr-3Mo-1Cr [53]	α + β	1048 (UTS) 1001 (YS)	-	20.3	-	β + α/β forging + double annealing 910 °C/1 h, FC + 590 °C/4 h, AC
Ti-5.5Cr-5Al-4Mo-3Nb-2Zr [54]	β	1228 ± 13 (UTS) 1159 ± 17 (YS)	-	11.3	-	Vacuum arc melting 850 °C/0.5 h, WC + 650 °C/6 h, AC
Ti-5Al-4Zr-8Mo-7V [55]	β	1499 (UTS) 1436 (YS)	-	10.8	-	β-transus forging 760 °C/3 h FC + 600 °C/8 h AC
Ti-5Al-5Mo-5V-3Cr-1Zr [56]	Near-β	1397 ± 15 (UTS) 1312 ± 16 (YS)	-	5.7 ± 0.1	-	multi-stage isothermal forging 1100/950/910/(Tβ + 15) °C multi-pass forging
Ti-10V-2Fe-3Al [57]	Near-β	1180 (UTS) 1090 (YS)	-	13	-	β + α/β forging 870 °C/2.5 h FC → 700 °C AC + 570 °C/6 h AC
Ti-5Al-2Sn-2Zr-4Mo-4Cr [58]	Near-β	1303 (UTS) 1276 (YS)	-	7.01	-	α + β hot rolling + single aging 860 °C rolling (40% reduction) + 540 °C/8 h, AC
Ti-7Mo-3Nb-3Cr-3Al [59]	Near-β	1410 (UTS) 1230 (YS)	-	8.6	-	Multi-directional forging + STA 820 °C/30 min WQ + 525 °C/8 h AC

**Table 2:** Recommended microstructures for different service conditions.

Service Condition	Examples	Microstructure	Rationale
High-temperature load bearing	Turbine discs, casings	Lamellar or near-lamellar	Creep resistance [85,86,87,88,89,90]; crack deflection [84]; reduced grain-boundary sliding [81].
Cyclic loading	Airframe structures, landing gears	Equiaxed or fine bimodal	Homogeneous strain partitioning [93]; delayed crack initiation [37].
Comprehensive performance	Complex structural parts	Bimodal	Hetero-deformation strengthening [37]; delayed local instability [91].
Ultra-high strength	Fasteners, high-strength inserts	Metastable β	Stress-induced transformation/twinning provides extra work hardening [95,100].
Room-temperature high ductility	Sheet metal, cryogenic components	Equiaxed	Maximum slip activation [93]; lowest strain localisation [37].

**Table 3:** Various twinning systems in α-Ti (hcp) [122].

Twinning Plane (K_1_)	Misorientation (Angle/Axis)	Shear Strain (s)	Type in Ti (c/a < 1.633)
{10–12}	85.0° [11–20]	0.175	Extension
{10–11}	57.2° [11–20]	0.343	Contraction
{11–22}	64.4° [1–100]	0.152	Contraction
{11–21}	35.0° [10–10]	0.515	Extension

## Data Availability

No new data were created or analyzed in this study. Data sharing is not applicable to this article.
